# Characterization of Lignin-Phenol-Formaldehyde Resins Synthesized with Different Lignin Sources

**DOI:** 10.3390/polym18111313

**Published:** 2026-05-26

**Authors:** Hyeon Ji Im, Sa Rang Choi, Jung Myoung Lee

**Affiliations:** Department of Wood and Paper Science, Kyungpook National University, 80 Daehakro, Daegu 41566, Republic of Korea; hyeonji1872@naver.com (H.J.I.); luvvchoi@knu.ac.kr (S.R.C.)

**Keywords:** lignin, phenolic hydroxyl group, lignin-phenol-formaldehyde resin, plywood, sustainable resin

## Abstract

The growing demand for sustainable adhesives has increased interest in lignin as a renewable alternative to phenol in PF resins. In this study, lignins from hardwood, softwood, and bamboo were evaluated as partial replacements for phenol in plywood and paper panel applications. Softwood lignin exhibited the highest molecular weight and guaiacyl-rich structure, hardwood lignin contained more syringyl units, and bamboo lignin presented notable p-hydroxyphenyl content with minimal ash and sulfur. Incorporating up to 50% lignin increased resin viscosity and markedly shortened gel time from 136–152 s for neat PF resins to 58–139 s, depending on lignin type and resin formulation. Among the tested lignins, softwood lignin produced the strongest adhesives, whereas bamboo lignin resulted in the lowest formaldehyde emission (<0.6 mg/L). These findings demonstrate the feasibility of tailoring LPF resin performance by selecting lignin type.

## 1. Introduction

Phenol-formaldehyde (PF) resin, a thermosetting polymer synthesized through the condensation reaction of phenol and formaldehyde, has a wide range of industrial applications [1,2,3]. Particularly, this material is used as an adhesive in wood-based products such as plywood, particleboard, and fiberboard, playing a crucial role in the industrial and construction sectors. Despite its strong adhesive properties, PF resin presents several challenges due to its reliance on petroleum-based raw materials. Petroleum resources are finite, and their extraction and processing contribute to environmental issues, including global warming. Additionally, PF resins release carcinogenic formaldehyde gas during use, posing a serious threat to human health. Given these concerns, there is growing interest in exploring environmentally friendly, economically viable alternatives to petroleum-based raw materials while assessing their feasibility for practical applications [4].

Numerous studies have investigated bio-based adhesives to reduce dependence on fossil fuels, focusing on sustainable and innovative raw materials such as lignin, starch, and tannin [5,6,7]. Lignin, a natural phenolic polymer, is abundant in the cell walls of both woody and non-woody plants. It inherently acts as a natural adhesive, binding cellulose and hemicellulose to enhance structural stability [8]. Lignin is primarily extracted as a by-product of pulp production [9]. The Kraft pulping method, the most common, has several significant drawbacks, including high energy costs and the environmental impact of sulfur use. Unlike kraft pulping, organosolv pulping provides sulfur-free lignin and retains a portion of carbohydrate-derived components, resulting in lignin with structural features and reactivity distinct from conventional kraft lignin. These characteristics influence its solubility, reactivity, and performance in resin synthesis. Lignin has become increasingly recognized across various fields, and recent research has focused on enhancing its value as a polymer by leveraging its structural properties [10]. Notably, lignin is being re-evaluated as a viable substitute for phenol in PF resins, offering a more sustainable approach to resin synthesis [11,12,13]. Lignin consists of three monomers (coniferyl alcohol, sinapyl alcohol, and p-coumaryl alcohol), which undergo enzymatic polymerization to form monolignol units [14]. These monolignols form three fundamental types of phenylpropane units: syringyl, guaiacyl, and p-hydroxyphenyl. Softwood lignin primarily consists of guaiacyl and p-hydroxyphenyl units. In contrast, hardwood lignin contains a higher proportion of syringyl units and a relatively lower amount of guaiacyl units than softwood lignin. Interestingly, despite being derived from a non-woody species, bamboo lignin shares a similar composition with woody lignin, comprising syringyl, guaiacyl, and p-hydroxyphenyl units. However, bamboo lignin has a higher concentration of phenolic hydroxyl (OH) groups than woody lignin [15]. This increased abundance of phenolic OH groups enhances its reactivity during pulping and other pretreatment processes, making it more responsive to chemical modifications [16]. The unique structural features of lignin, particularly its phenolic OH groups, facilitate its reaction with formaldehyde in resin synthesis. Unlike hardwood lignin, softwood lignin readily reacts with formaldehyde due to its open ortho positions and the predominance of guaiacyl and p-hydroxyphenyl units [17]. Therefore, various methods have been explored to enhance the reactivity of the relatively less reactive hardwood lignin. One study investigated a technique that involved adjusting the pH of black liquor from softwood and hardwood, followed by fractionation of the precipitated lignin at the adjusted pH [18]. Additionally, experiments on the synthesis of lignin-phenol-formaldehyde (LPF) resins have been conducted to evaluate strategies to improve the reactivity of hardwood lignin [19].

In addition to pH control, various methods, such as solvent extraction, membrane separation, and solvent-water extraction, have been developed to achieve more uniform lignin extraction from black liquor [20]. Among these, solvent–water extraction is particularly effective for fractionating lignin, resulting in a more homogeneous chemical structure and enhancing its potential for valorization. Furthermore, several techniques, including demethylation and phenolation, have been developed to enhance lignin reactivity. While demethylation effectively increases the number of reactive sites, it is a complex and costly process [21]. In contrast, phenolation offers a relatively simple and efficient means of enhancing lignin’s reactivity [22,23]. Extensive research on lignin as a partial phenol substitute has shown that its reactivity is strongly influenced by structural characteristics, including biomass origin, extraction method, and phenolic hydroxyl content. These factors determine lignin’s reactivity with formaldehyde, thereby affecting the performance of lignin–phenol–formaldehyde (LPF) resins. However, despite substantial research, a systematic comparison of lignin sourced from different biomass types, along with an examination of how these differences influence LPF resin performance in both veneer bonding and paper impregnation applications, remains limited. In particular, resin systems used for plywood (water-based PF) and paper laminates (alcohol-based PF) require different molecular characteristics. Water-based PF resins typically exhibit high viscosity and slower curing rates, whereas methanol-based PF resins require low-molecular-weight components to achieve rapid penetration and faster curing. These differences in processing requirements suggest that performance may vary with lignin type, necessitating comparisons across lignin types based on their structures and resin synthesis methods. Although numerous studies have investigated lignin substitution in PF resins, direct comparisons of lignins derived from different biomass origins under multiple industrially relevant LPF resin systems remain limited. In particular, the relationships between lignin structure, resin curing behavior, adhesive performance, and formaldehyde emission characteristics have not been systematically compared for hardwood, softwood, and bamboo lignins under identical formulation conditions.

Therefore, in this study, various lignin-based phenolic resins (hardwood and softwood technical lignin, bamboo organosolv lignin) were synthesized using two phenolic resin synthesis routes typically employed for veneer and paper bonding, and the fundamental properties of lignin and the performance of the resulting resins were evaluated. Previous studies have reported that lignin substitution begins to exert a more noticeable influence on resin properties at replacement levels above approximately 20% [24]. In addition, owing to the relatively low reactivity and heterogeneous structure of lignin, unmodified lignin is generally used at substitution levels up to 30–50% in PF resins without significant deterioration of resin performance, whereas higher substitution levels may negatively affect curing behavior and adhesive properties [25,26]. Therefore, 30% and 50% substitution levels were selected in this study to evaluate the balance between lignin incorporation and resin performance. The performance evaluation of all synthesized resins was conducted using veneer-based testing. Elemental composition and molecular weight distribution were examined to characterize the chemical features of each lignin, and the phenolic hydroxyl content was evaluated to understand lignin’s structural attributes and reactivity toward formaldehyde. To assess the feasibility of substituting phenol with lignin in PF resins, lignin–phenol–formaldehyde (LPF) resins were synthesized via both resin preparation routes, and their properties, together with the bonding performance of the resulting plywood specimens, were evaluated.

## 2. Materials and Methods

### 2.1. Materials

The hardwood technical kraft lignin (HL) used in this study was sourced from Moorim P&P Co., Ltd. (Ulsan, Republic of Korea), while the softwood technical kraft lignin (SL) was obtained from Stora Enso (Helsinki, Finland). Bamboo-based non-woody lignin (BL) was isolated from bamboo black liquor through acid-catalyzed organosolv bamboo pulping. Paraformaldehyde (95%, Extra Pure) and phenol (98%, Extra Pure) were purchased from Duksan Pure Chem. Co., Ltd. (Ansan, Gyeonggi-do, Republic of Korea) for resin synthesis, and NaOH was used as the catalyst. The wood used in plywood manufacturing was provided by Daejung Chemical Co., Ltd. (Siheung, Gyeonggi-do, Republic of Korea).

### 2.2. Extraction of Bamboo-Based Organosolv Lignin

A mixed solvent was prepared by combining an organic solvent and distilled water in a 1:1 (*v*/*v*) ratio, followed by the addition of sulfuric acid to achieve a final concentration of 0.4 M relative to the total volume of the solution. Bamboo wood chips (300 g) were immersed in the prepared solution at a liquor-to-wood ratio of 3:1 (*w*/*w*) for 12 h. During the impregnation process, a vacuum chamber was used to completely remove air from the chips, thereby facilitating thorough penetration of the solution into the wood matrix. Pulping was conducted in an autoclave (HST 506-6, Hanbaek ST, Bucheon, Gyeonggi-do, Republic of Korea) at 120 °C for 6 h, and the resulting acid black liquor was separated from the solid residue by filtration. To precipitate lignin from the acid black liquor, the liquor was diluted with distilled water at a 1:4 (*v*/*v*) ratio and stirred for 30 min to promote lignin precipitation. The precipitated lignin was collected by vacuum filtration and washed repeatedly with distilled water until the filtrate reached neutrality.

### 2.3. Synthesis of LPF Resins

LPF resins were synthesized from paraformaldehyde (F) and phenol (P) at an F/P molar ratio of 1.3, based on the molecular weights and purities of the reactants. Lignin was added at 30% and 50% of the total dry weight of phenol, while maintaining the F/P molar ratio. Sodium hydroxide, applied at a NaOH/P molar ratio of 0.3, was used as the catalyst. The detailed composition is presented in Table 1. The mixture was heated to 95 °C and maintained for 3 min, after which the temperature was rapidly reduced to 65 °C to promote controlled condensation. The reaction was then continued for 1 h. Depending on the intended application, two types of resin were prepared. The plywood adhesive was used directly after synthesis without additional dehydration, whereas the paper-panel resin underwent a dehydration step following synthesis. The solids content of the dehydrated paper-panel resin was subsequently adjusted by adding methanol to replace the water removed.

### 2.4. Fabrication of Plywood

Veneers measuring 300 mm × 300 mm with a moisture content of approximately 9% were used without additional drying. To adjust the viscosity of the adhesive mixture, 10% food-grade flour was incorporated into the resin formulation. The mixture was then applied at a spread rate of 180 g/m^2^ to prepare 3-ply plywood. The assembled veneers were first cold-pressed at 1.4 MPa for 20 min, followed by hot-pressing at 1.4 MPa and 170 °C for 4 min. After pressing, the plywood panels were conditioned at 25 °C for at least 24 h prior to testing for tensile shear strength and formaldehyde emission.

### 2.5. Evaluation of Lignin Properties

#### 2.5.1. Total Lignin and Elemental Analysis

To determine the Klason lignin (KL) content, lignin samples were immersed in 72% H_2_SO_4_ for 12 h, after which the acid concentration was diluted to 3%. The lignin was then hydrolyzed at 120 °C for 2 h. Thereafter, the resultant liquid was filtered under vacuum, and the residue was dried and weighed. Acid-soluble lignin (ASL) was quantified by collecting and diluting the filtrate from the Klason lignin filtration step and measuring absorbance at 205 nm using a UV/VIS spectrophotometer (Optizen 3220UV, Mecasys Co., Ltd., Daejeon, Republic of Korea). Elemental analysis of lignin was performed using a FlashSMART Elemental Analyzer (ThermoFisher, Waltham, MA, USA). All measurements for total lignin and elemental analysis were conducted in triplicate, and the results are presented as mean ± standard deviation.

#### 2.5.2. Gel Permeation Chromatography (GPC) Analysis

To determine the molecular weight of lignin, acetylation was performed. Specifically, 100 mg of lignin was reacted with 2 mL of pyridine and 2 mL of acetic anhydride for 12 h to ensure complete dissolution. The resulting solution was precipitated in ice water, and the precipitated lignin was filtered through a 0.45 μm nylon membrane before vacuum-drying for analysis. The presence of acetyl groups was confirmed via Fourier-transform infrared spectroscopy [27]. For GPC analysis, 2 mg of acetylated lignin was dissolved in 1 mL of tetrahydrofuran. The analysis was conducted at a flow rate of 1 mL/min, with measurements recorded at 35 °C over a 45 min period using a Waters Alliance e2695 HPLC system (Milford, MA, USA).

#### 2.5.3. Attenuated Total Reflectance Infrared (ATR-IR) Spectroscopy

All lignin samples were characterized using ATR-IR spectroscopy (Bruker Optics, Ettlingen, Germany). Spectral data were collected over 32 scans in the range of 4000–400 cm^−1^, with a resolution of 4 cm^−1^. ATR-IR spectra were obtained from three independent measurements for each lignin sample.

#### 2.5.4. ^31^P Nuclear Magnetic Resonance (NMR) Spectroscopy

Dried lignin (20 mg) was completely dissolved in a solvent mixture prepared for ^31^P NMR analysis. The solvent system consisted of deuterated chloroform and anhydrous pyridine (1:1.6, *v*/*v*), supplemented with chromium (III) acetylacetonate (Cr(acac)_3_, 5.0 mg/mL) as a relaxation agent and N-hydroxy-5-norbornene-2,3-dicarboximide (NHND, 18 mg/mL) as an internal standard. To the lignin solution, 0.05–0.1 mL of 2-chloro-4,4,5,5-tetramethyl-1,3,2-dioxaphospholane (TMDP) was added as the phosphitylation reagent. The ^31^P NMR spectra were acquired on a 500 MHz NMR spectrometer (Bruker Avance III 500, Ettlingen, Germany) using 512 scans, a 90° pulse angle, a 10 s relaxation delay, and an acquisition time of 0.8 s. ^31^P NMR analysis was conducted using independently prepared samples for each lignin type.

### 2.6. Characterization of the PF and LPF Resins

The properties of the synthesized LPFs were evaluated to determine the effect of lignin incorporation. The solids content was measured by drying 2 g of resin in an oven at 105 °C for 3 h and determining the dry weight relative to the initial weight. Density was measured as the weight per unit volume using 100 mL of resin. Viscosity was assessed using a cone plate viscometer (DV-II+, Brookfield, Middleboro, MA, USA) with spindle 82 at 20 rpm and 25 °C [28]. Gelation time was determined on a hot plate at 150 °C. All resin property measurements were conducted at least in triplicate. All resin property measurements were conducted at least in triplicate. Statistical analysis was performed using one-way analysis of variance (ANOVA) followed by Tukey’s post hoc test using OriginPro 2024 software (OriginLab Corporation, Northampton, MA, USA), and differences were considered significant at *p* < 0.05.

### 2.7. Characterization of 3-Plywood

Tensile strength was measured using a universal testing machine (H50KS, Hounsfield, Redhill, UK) at a crosshead speed of 2 mm/min, using twelve specimens measuring 80 mm × 25 mm. For formaldehyde emission testing, twelve specimens (15 cm × 5 cm) were placed in a desiccator containing 300 mL of distilled water and maintained at 20 °C for 24 h in accordance with KS F 3101. The absorbed formaldehyde in the water was then reacted with an acetylacetone–acetic acid–ammonium solution, heated at 65 °C for 10 min, cooled, and subsequently analyzed. Formaldehyde concentration was determined using a UV/VIS spectrophotometer (Optizen 3220UV, Mecasys Co., Ltd., Daejeon, Republic of Korea) at 412 nm, following the Japanese Industrial Standard (JIS A 1460) [29]. All measurements were conducted using twelve replicate specimens, and the results are presented as mean ± standard deviation. All measurements were conducted using at least twelve replicate specimens, and the results are presented as mean ± standard deviation.

## 3. Results and Discussion

### 3.1. Lignin Purity and Composition Analysis

**Figure 1:**
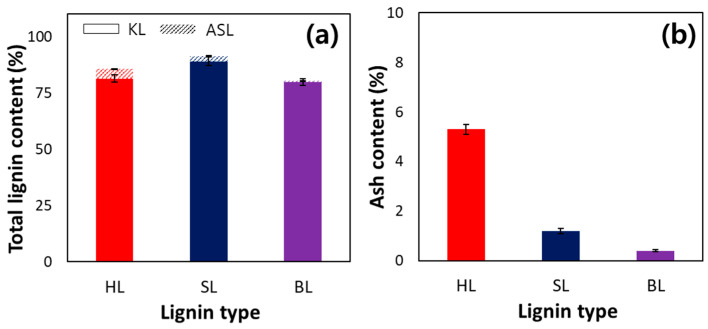
Purity of lignins derived from different sources (**a**) Total lignin content composed of Klason lignin (KL) and acid-soluble lignin (ASL), and (**b**) ash content.

The purity of the three lignin samples (HL, SL, and BL) was evaluated based on their total lignin and ash contents. As shown in Figure 1a, HL and SL, both classified as technical lignins, exhibited the high total lignin contents of 85.6% and 91.4%, respectively. In contrast, BL, which was extracted from organosolv-derived black liquor, showed a relatively lower total lignin content of 80.4%. During the organosolv pulping process, the acid catalyst not only depolymerizes lignin but also hydrolyzes hemicellulose, generating low molecular weight carbohydrate derivatives such as furfural [30]. These carbohydrate-derived compounds may co-precipitate with lignin during recovery or remain incompletely removed after extraction, thereby acting as non-lignin components that lower the overall lignin purity.

The ash content, which represents the amount of inorganic residue in the lignin samples, is shown in Figure 1b. HL exhibited the highest ash content (approximately 5.3%), which can be attributed to residual inorganic chemicals used in the kraft pulping process. In contrast, SL and BL showed ash contents of approximately 1.3% and 0.4%, respectively. The low ash content of BL reflects the characteristic advantage of organosolv pulping, in which minimal inorganic residues remain [31], suggesting that BL can reduce adverse effects caused by inorganic impurities during resin synthesis.

Elemental analysis (Table 2) revealed that SL and BL exhibited higher carbon (C) contents than HL. Moreover, BL showed a sulfur (S) content of 0.0%, clearly distinguishing it from HL (1.4%) and SL (1.7%), both of which are produced through the Kraft process. The absence of sulfur offers advantages in reducing odor and corrosion problems in resin products. Therefore, although BL exhibited the lowest total lignin content, its extremely low sulfur and ash contents indicate favorable characteristics for minimizing impurity-related interference during resin synthesis.

### 3.2. Molecular Weight Distribution of Lignins

Gel permeation chromatography (GPC) was used to analyze the molecular weight distribution of each lignin type. The number-average molecular weight (Mn), weight-average molecular weight (Mw), and polydispersity index (PDI) are presented in Table 3. HL exhibited the lowest molecular weights among the three lignin samples, with a number-average molecular weight of 2680 g/mol and a weight-average molecular weight of 4877 g/mol. In contrast, SL showed the highest molecular weights (3370 g/mol and 8602 g/mol), whereas BL (3226 g/mol and 6056 g/mol) fell within the intermediate range between HL and SL. The polydispersity index (PDI) represents the uniformity of lignin molecular size distribution. All lignin samples exhibited PDIs below 3, indicating relatively narrow molecular weight distributions. SL showed the broadest distribution (PDI = 2.60), suggesting that softwood kraft lignin possesses a relatively higher degree of structural heterogeneity. In contrast, HL (1.82) and BL (1.88) showed narrower distributions, implying more uniform molecular fractionation during extraction. The molecular weight of lignin is a key determinant of its reactivity with formaldehyde during LPF resin synthesis. Lower-molecular-weight lignin generally exhibits higher reactivity because the reactive functional groups, such as phenolic hydroxyls, are more readily accessible compared with those in higher-molecular-weight lignin [32]. Therefore, low molecular weight is typically advantageous for accelerating condensation reactions and promoting resin polymerization. Although lower molecular weight lignin tends to exhibit higher intrinsic reactivity toward formaldehyde, it is well known that additional factors, including ash content, sulfur-containing species, and structural heterogeneity [33], can significantly influence overall curing behavior. Thus, molecular weight alone does not fully determine lignin reactivity, and the final resin performance should be interpreted in conjunction with these complementary characteristics.

### 3.3. ATR-IR Spectroscopy of Lignin

**Figure 2:**
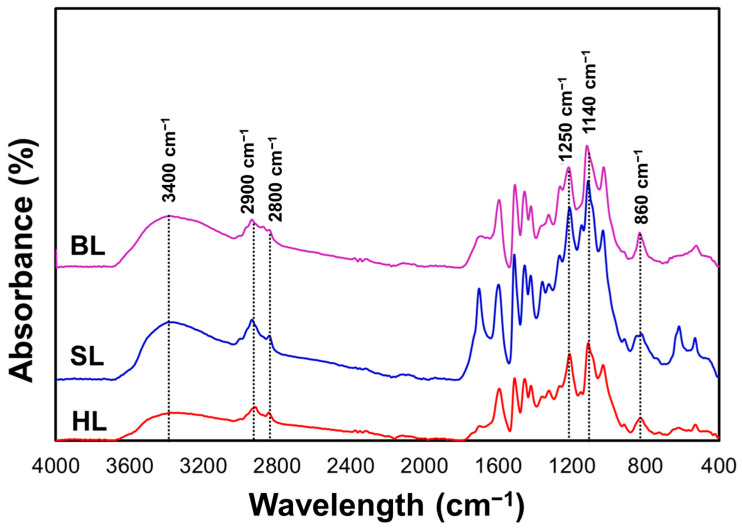
ATR-IR spectra of the three lignin samples.

The ATR-IR spectra of HL, SL, and BL are presented in Figure 2. All lignin samples exhibited a broad peak at approximately 3400 cm^−1^, corresponding to aromatic and aliphatic O–H groups. This band appeared wide and intense in the SL and BL samples, suggesting that SL and BL may possess a comparatively higher abundance of hydroxyl functionalities. All lignin samples exhibited distinct peaks in the 2900–2800 cm^−1^ region, associated with the C–H stretching of the aliphatic groups forming the lignin backbone [14,18,34]. In the aromatic skeletal region near 1600–1500 cm^−1^, SL displayed stronger absorbance than HL, reflecting a greater degree of aromatic condensation typically associated with guaiacyl (G)-rich softwood lignin. The most distinctive differences among the samples appeared in the 1250–1140 cm^−1^ region, which exhibited trends consistent with the lignin classification framework proposed by Faix (1991) [35], differentiating G-type (softwood) and GS-type (hardwood) lignins. The relative intensities of the bands at 1140 cm^−1^ assigned to aromatic C–H in-plane deformation and 1250 cm^−1^ associated with C–C and C–O stretching of guaiacyl units, with additional contributions from C=O stretching, provide a useful diagnostic metric for distinguishing lignin types. In the softwood-derived SL sample, the 1140 cm^−1^ band was substantially more intense than the 1250 cm^−1^ band. This behavior can be explained by the highly condensed structure typically formed during kraft pulping and by the absence of syringyl (S) units, both of which diminish the contribution of phenolic C–O stretching vibrations in the 1250 cm^−1^ region. In contrast, the hardwood-derived HL sample displayed nearly equivalent intensities at 1250 and 1140 cm^−1^. This reflects the influence of syringyl units, which are more highly methoxylated and dilute the guaiacyl-specific spectral features, producing the characteristic GS-type pattern observed for hardwood lignins. Additional distinctions were observed near 860 cm^−1^, corresponding to aromatic C–H out-of-plane deformation. The position and magnitude of this band are known to depend on the substitution patterns of G, S, and H units. As reported by Faix [35], HGS-type lignins exhibit a pronounced absorption around 834 cm^−1^ due to contributions from p-hydroxyphenyl (H) units. Consistent with this behavior, the BL sample showed a noticeably stronger peak in the 860 cm^−1^ region than both SL and HL, indicating a significant presence of H units in bamboo lignin in addition to G and S structures.

### 3.4. ^31^P NMR Spectroscopy for Determining OH Group Content

^31^P NMR spectroscopy is one of the most powerful techniques for quantitatively determining the contents of aliphatic, phenolic, and carboxylic hydroxyl groups in lignin (Table 4). The aliphatic hydroxyl groups, primarily located on the lignin side chains, were present at similarly high levels in SL (1.76 mmol/g) and BL (1.71 mmol/g), whereas HL showed a considerably lower value (0.83 mmol/g). The reduced aliphatic hydroxyl content in HL suggests that side-chain oxidation may have occurred to a greater extent during its production or extraction process, or that the original structural characteristics indicate a modification may have occurred at the γ-carbon position of the side chain [14,36].

Clear distinctions among the samples were also observed in the distribution of phenolic hydroxyl groups, a key determinant of lignin type. In the SL sample, S–OH (syringyl hydroxyl) groups were not detected, while G–OH (guaiacyl hydroxyl) accounted for nearly all phenolic hydroxyls (1.52 mmol/g). This result is consistent with the literature, which describes softwood lignin as being composed almost exclusively of guaiacyl units [37], and it supports the FTIR observations of a weak 1250 cm^−1^ band and a strong 1140 cm^−1^ band. In HL, the S–OH content (1.63 mmol/g) exceeded that of G–OH (1.00 mmol/g), yielding an S/G ratio of approximately 1.63. HL also exhibited the highest total phenolic hydroxyl content among the samples, at 2.86 mmol/g. The BL sample displayed a significantly higher amount of H–OH (p-hydroxyphenyl hydroxyl) groups (0.39 mmol/g) compared with SL (0.15 mmol/g) and HL (0.23 mmol/g). This finding aligns with previous reports indicating that herbaceous lignins, such as those from bamboo, contain notable amounts of H-type units derived from p-coumarate structure [38].

### 3.5. Properties of the LPF Resins

HL, SL, and BL lignins were used to replace 30% and 50% of the phenol in the synthesis of LPF resins. Two types of resins were prepared one formulated for plywood applications and the other for paper-impregnation use. In addition, a conventional phenol–formaldehyde resin without lignin substitution (0% PF) was synthesized as the control. The densities of all synthesized resins were in the range of 1.10–1.14 g/mL, showing values similar to those of the control PF resin (1.12 and 1.10 g/mL) (Table 5). This similarity indicates that the incorporated lignin was uniformly integrated into the phenol–formaldehyde polymer network without causing noticeable changes in bulk resin density.

**Figure 3:**
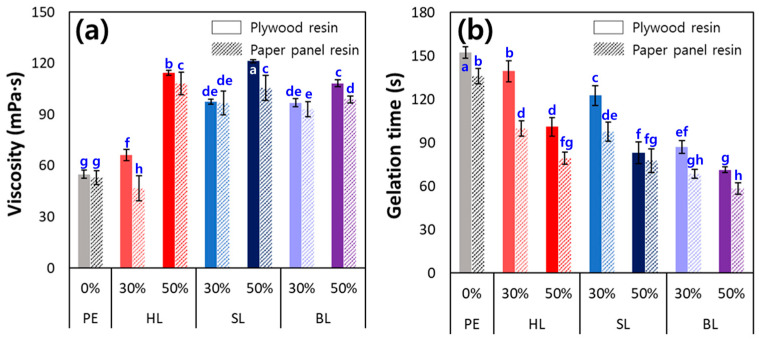
Comparison of (**a**) viscosity and (**b**) gelation time in LPF resins for plywood and paper panel applications. Different lowercase letters indicate statistically significant differences among samples (*p* < 0.05).

To evaluate the processability and curing behavior of the synthesized LPF resins, viscosity and gel time were measured, and the results are presented in Figure 3. As shown in the viscosity analysis (Figure 3a), all LPF resins with 30% and 50% lignin replacement exhibited higher viscosities than the control PF resin (0% PF). Moreover, the viscosity increased noticeably as the lignin substitution increased from 30% to 50%. This increase in viscosity is due to the macromolecular structure of lignin, which, compared to phenol, is a single molecule. This macromolecular structure increases the average molecular weight during resin synthesis. Furthermore, lignin’s complex molecular structure is interpreted to increase intermolecular friction within the solution, thereby reducing fluidity [39,40]. Among the samples, the SL-based resins showed the highest viscosities. This is consistent with the structural characteristics of softwood lignin, which form a highly condensed guaiacyl-rich network, leading to larger, more rigid macromolecules. These observations align with previous reports indicating that higher lignin content and larger lignin molecular weight are associated with increased resin viscosity [14].

Gel time, which reflects the reactivity of the resin (shorter times indicating faster curing) is shown in Figure 3b. The control resin (0% PF) exhibited the longest gel times (136 and 152 s), whereas all lignin-containing resins displayed significantly shorter gel times of 58–139 s. This inverse relationship between gel time and the extent of polycondensation or cross-linking has also been noted in previous studies [41]. Notably, increasing the lignin substitution from 30% to 50% resulted in a substantial reduction in gel time. Because lignin has a larger molecular framework than phenol, greater lignin incorporation may influence the bulk gelation behavior of the resin system, leading to faster gel formation. Overall, while lignin incorporation increases resin viscosity, it markedly accelerates curing, potentially reducing processing time in industrial applications. In addition, the LPF resins formulated for paper impregnation exhibited lower viscosities and shorter gel times than those formulated for plywood applications. This difference is likely attributable to the relatively low boiling point of the methanol used to control the solids content during dehydration, which influences subsequent gelation behavior. The interpretations regarding curing behavior and network formation were primarily based on bulk viscosity and gel time measurements. Therefore, further thermal and rheological analyses, such as DSC or rheological measurements, would be beneficial for a more detailed understanding of LPF curing behavior.

### 3.6. Properties of the Plywood Prepared Using the LPF Resins

**Figure 4:**
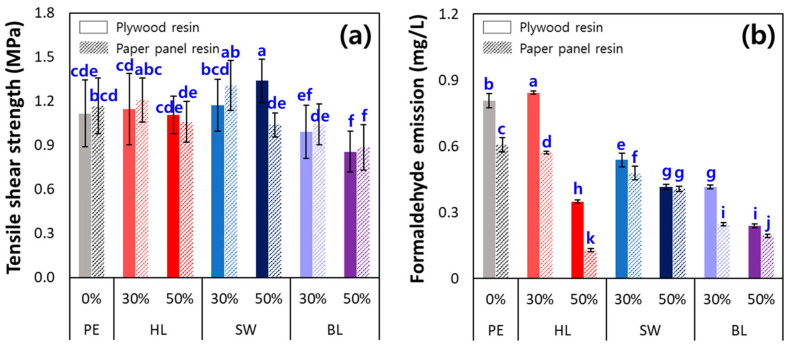
Comparison of (**a**) tensile shear strength and (**b**) formaldehyde emission in LPF-formulated plywood and paper panels. Different lowercase letters indicate statistically significant differences among samples (*p* < 0.05).

The tensile strength and formaldehyde emissions of plywood prepared using the synthesized LPF resins are presented in Figure 4. The LPF resins synthesized with 30% and 50% phenol replacement for HL and SL exhibited adhesion strengths comparable to those of the 0% PE resin. Notably, for both plywood and paper panels, the tensile strength of SL-based LPF resin was higher than that of HL-based resin. The high viscosity and rigidity observed in the SL-based resins can be attributed to the structural characteristics of softwood lignin. According to Ghorbani et al. (2016) [42] and Tejado et al. (2007) [14], softwood lignin is predominantly composed of guaiacyl (G) units, which form highly condensed structures through extensive C–C linkages. This structural rigidity limits the mobility of polymer chains during resin synthesis, thereby increasing viscosity. After curing, the condensed G-unit network promotes the formation of a denser, more rigid three-dimensional crosslinked structure, which, in turn, enhances the mechanical strength and thermal stability of the resulting resin. BL-based LPF resin showed the lowest bonding strength. This is believed to be due to the low purity of bamboo lignin. Zhang et al. (2013) [39] reported that higher carbohydrate content resulted in lower strength and water resistance of the resin because carbohydrates present in lignin reduced its reactivity.

Despite this, the BL-based resin exhibited the lowest overall formaldehyde emissions. Additionally, resins with viscosities exceeding 100 mPa·s exhibited overall low formaldehyde emissions of approximately 0.6 mg/L or less. Highly viscous resins tend to form a denser three-dimensional network upon curing, allowing them to more effectively capture free formaldehyde. This is achieved either by chemically bonding reactive functional groups, particularly phenolic hydroxyl groups, to methylene bridges or by physically trapping unreacted molecules within the polymer matrix. Consequently, increasing viscosity and crosslinking density reduces the amount of formaldehyde released into the environment [43]. Furthermore, the LPF resin, formulated for paper panel and undergoing a dehydration step, showed lower formaldehyde emissions than the plywood resin. Removal of moisture shifts the curing equilibrium toward the methylene bond, thereby promoting more complete incorporation of formaldehyde into the polymer network. This reduces the concentration of emittable free formaldehyde [44].

The results suggest that the structural characteristics and purity of lignin influence the curing behavior, viscosity, and bonding performance of LPF resins. In particular, differences in lignin structure affected resin viscosity, gelation behavior, tensile shear strength, and formaldehyde emission characteristics depending on the lignin source. These findings indicate that lignin type should be carefully considered when designing LPF resins for specific applications. Although the present study focused on the initial bonding performance and formaldehyde emission properties of LPF resins, further evaluation of long-term durability, including moisture resistance, thermal stability, and aging behavior, will be necessary for broader industrial applications.

## 4. Conclusions

This study evaluated hardwood, softwood, and bamboo lignins as partial phenol substitutes for lignin–phenol–formaldehyde (LPF) resins via plywood and paper-impregnation applications. The lignin sources showed distinct differences in molecular weight, hydroxyl group distribution, and inorganic content, which influenced the properties of the resulting LPF resins. Incorporation of lignin increased resin viscosity and reduced gel time compared with neat PF resin. Among the tested lignins, softwood lignin produced resins with the highest tensile shear strength, whereas bamboo lignin showed the lowest formaldehyde emission despite its relatively low purity. The results indicate that lignin type affects the curing behavior and bonding performance of LPF resins and should be considered when selecting lignin feedstocks for phenolic resin applications.

## Figures and Tables

**Table 1:** Formulation details of PE and LPF resins with varying lignin.

Sample	Oven-Dry Weight (g)				Note
	Formaldehyde	Phenol	NaOH	Lignin	
0% PE	62.23	150.00	19.15	0.00	No lignin
30% HL	49.79	120.00	15.32	36.00	Hardwood lignin
30% SL					Softwood lignin
30% BL					Bamboo lignin
50% HL				60.00	Hardwood lignin
50% SL					Softwood lignin
50% BL					Bamboo lignin

**Table 2:** Elemental composition of hardwood, softwood, and bamboo lignins.

Lignin Type	Elemental Analysis				
	C (%)	H (%)	N (%)	S (%)	O (%)
HL	59.0 ± 0.08	5.3 ± 0.05	0.2 ± 0.02	1.4 ± 0.04	34.1 ± 0.26
SL	63.0 ± 0.18	5.6 ± 0.01	0.1 ± 0.01	1.7 ± 0.02	29.6 ± 0.39
BL	62.0 ± 0.04	5.9 ± 0.02	0.4 ± 0.01	0.0 ± 0.00	31.7 ± 0.01

**Table 3:** Results of the GPC analysis of lignin.

Lignin Type	Mn (g/mol)	Mw (g/mol)	PDI (Mw/Mn)
HL	2680	4877	1.82
SL	3370	8602	2.60
BL	3226	6056	1.88

**Table 4:** ^31^P NMR analysis results (mmol/g lignin) of different lignin samples.

Lignin Type	Aliphatic OH (mmol/g)	Aromatic OH (mmol/g)				COOH (mmol/g)
		S-OH	G-OH	H-OH	Total Phenolics	
HL	0.83	1.63	1.00	0.23	2.86	2.86
SL	1.76	-	1.52	0.15	1.67	0.26
BL	1.71	0.51	0.69	0.39	1.59	0.34

**Table 5:** Density of PE and LPF resins prepared for plywood and paper panel applications.

LPF	Density (g/mL)	
	Plywood Resin	Paper Panel Resin
0% PE	1.12 ± 0.00	1.10 ± 0.00
30% HL	1.13 ± 0.00	1.13 ± 0.00
50% HL	1.14 ± 0.00	1.12 ± 0.00
30% SL	1.12 ± 0.01	1.10 ± 0.00
50% SL	1.13 ± 0.00	1.12 ± 0.00
30% BL	1.12 ± 0.00	1.10 ± 0.00
50% BL	1.12 ± 0.00	1.11 ± 0.00

## Data Availability

The original contributions presented in this study are included in the article. Further inquiries can be directed to the corresponding author.

## Abbreviations

The following abbreviations are used in this manuscript:

ASL	Acid-soluble lignin
ATR-IR	Attenuated total reflectance infrared spectroscopy
BL	Bamboo lignin
COOH	Carboxylic acid group
F	Formaldehyde
FTIR	Fourier-transform infrared spectroscopy
G–OH	Guaiacyl hydroxyl group
GPC	Gel permeation chromatography
H–OH	p-hydroxyphenyl hydroxyl group
HL	Hardwood lignin
KL	Klason lignin
LPF	Lignin–phenol–formaldehyde
Mn	Number-average molecular weight
Mw	Weight-average molecular weight
NHND	N-hydroxy-5-norbornene-2,3-dicarboximide
P	Phenol
PDI	Polydispersity index
PF	Phenol–formaldehyde
